# Roles of Peroxisome Proliferator-Activated Receptor α in Bitter Melon Seed Oil-Corrected Lipid Disorders and Conversion of α-Eleostearic Acid into Rumenic Acid in C57BL/6J Mice

**DOI:** 10.3390/nu8120805

**Published:** 2016-12-12

**Authors:** Ya-Yuan Chang, Hui-Min Su, Szu-Han Chen, Wen-Tsong Hsieh, Jong-Ho Chyuan, Pei-Min Chao

**Affiliations:** 1 Department of Nutrition, China Medical University, Taichung 404, Taiwan; i30489@hotmail.com (Y.-Y.C.); jasminne1117@hotmail.com (S.-H.C.); 2 Graduate Institute of Physiology, National Taiwan University, Taipei 100, Taiwan; hmsu1203@ntu.edu.tw; 3 School of Medicine, China Medical University, Taichung 404, Taiwan; wthsieh@mail.cmu.edu.tw; 4 Hualien District Agricultural Research and Extension Station, Hualien 973, Taiwan; jonghoc@mail.hdais.gov.tw

**Keywords:** PPARα, bitter melon seed oil, hepatic steatosis, obesity, α-eleostearic acid

## Abstract

We previously reported that bitter melon seed oil (BMSO) was an effective anti-steatosis and antiobesity agent. Since the major fatty acid α-eleostearic acid (α-ESA) in BMSO is a peroxisome proliferator-activated receptor α (PPARα) activator, the objective was to investigate the role of PPARα in BMSO-modulated lipid disorders and α-ESA metabolism. C57BL/6J wild (WD) and PPARα knockout (KO) mice were fed a high-fat diet containing BMSO (15% soybean oil + 15% BMSO, HB) or not (30% soybean oil, HS) for 5 weeks. The HB diet significantly reduced hepatic triglyceride concentrations and increased acyl-CoA oxidase activity in WD, but not in KO mice. However, regardless of genotype, body fat percentage was lowered along with upregulated protein levels of uncoupling protein 1 (UCP1) and tyrosine hydroxylase, as well as signaling pathway of cAMP-dependent protein kinase and AMP-activated protein kinase in the white adipose tissue of HB-treated groups compared to HS cohorts. In WD-HB and KO-HB groups, white adipose tissue had autophagy, apoptosis, inflammation, and browning characteristics. Without PPARα, in vivo reduction of α-ESA into rumenic acid was slightly but significantly lowered, along with remarkable reduction of hepatic retinol saturase (*RetSat*) expression. We concluded that BMSO-mediated anti-steatosis depended on PPARα, whereas the anti-adiposity effect was PPARα-independent. In addition, PPARα-dependent enzymes may participate in α-ESA conversion, but only have a minor role.

## 1. Introduction

Obesity is closely associated with an increased risk of nonalcoholic fatty liver disease (NAFLD); both conditions accelerate the pathological progress of type 2 diabetes and cardiovascular disease [1]. Excessive accumulations of triglyceride due to increased uptake or de novo lipogenesis, plus suppressed fatty acid oxidation or lipid export, in adipose and liver, are hallmarks of obesity and NAFLD. Consequently, functional lipids from dietary sources—including conjugated linoleic acid (CLA), phospholipids, and medium-chain triglycerides—that can modulate lipid metabolism and mitigate metabolic syndrome have attracted considerable attention [2,3].

Recently, we demonstrated that bitter melon seed oil (BMSO) is more potent than soybean oil, which has approximately the same amount of polyunsaturated fatty acid (PUFA), in attenuating high saturated fatty acid (SFA) diet-induced adiposity and hepatic steatosis in C57BL/6J mice [4,5]. BMSO is enriched in a fatty acid characterized by the presence of conjugated triene (i.e., *cis*-9, *trans*-11, *trans*-13 isomer of conjugated linolenic acid [6]), also termed α-eleostearic acid (α-ESA), which has potential as a functional lipid. Though α-ESA and its related isomer punicic acid (with *cis*-9, *trans*-11, *cis*-13 configuration) are converted efficiently into *cis*-9, *trans*-11 CLA (rumenic acid) in rats, mice, and humans [7,8,9,10], the enzymes enabling this delta-13 saturation reaction remain uncharacterized. Two nicotinamide adenine dinucleotide phosphate (NAD(P)H)-dependent enzymes, leukotriene B4 12-hydroxydehydrogenase/15-ketoprostaglandin delta 13-reductase (LTB4 12-HD/PGR), also known as prostaglandin reductase 1 (PTGR1) [7] and retinol saturase (RETSAT) [9], have been postulated, but never confirmed.

Based on a transactivation assay, α-ESA was a potent PPARα agonist compared to common fatty acids [11]. PPARα is a nuclear receptor that maintains lipid homeostasis by modulating an array of target genes participating in lipid catabolism, lipoprotein assembly and transport, as well as thermogenesis [12,13]. We previously demonstrated that α-ESA, as compared to α-linolenic acid (fatty acid control), reduced triglyceride concentrations in H4IIEC3, a PPARα-responsive hepatoma cell line [5]. In addition to activating PPARα directly, α-ESA activated sirtuin 1 by increasing the intracellular NAD^+^/NADH ratio, thus forming a positive feedback loop of PPARα/AMP-activated protein kinase (AMPK)/sirtuin 1 signaling [5].

PPARα activation may contribute to anti-adiposity by increasing fatty acid oxidation and thermogenesis [12,13]. We previously reported that the anti-adiposity effect of BMSO is associated with cAMP-dependent protein kinase (PKA) activation and cell death in white adipose tissue (WAT) [4]. In mice subjected to a BMSO diet, their WAT had delipidative, inflammatory, browning, apoptosis, and autophagy characteristics (determined by histochemistry combined with proteomic approaches), indicating a dynamic repair/remodel process [14]. In addition, α-ESA-induced apoptosis of 3T3-L1 preadipocytes may partially contribute to its anti-adipogenic activity [15].

Therefore, based on the importance of PPARα in systemic lipid homeostasis and α-ESA as a PPARα agonist, our objective was to investigate the role of PPARα in BMSO-mediated anti-steatosis and anti-adiposity functions. For this purpose, a 2 × 2 factorial design was used, with PPARα knockout (KO) and wild (WD) mice fed a high-fat diet composed of soybean oil alone (HS) or plus BMSO (HB). Parameters associated with lipid metabolism in liver and adipogenesis/remodeling in the WAT were determined. In addition, the role of PPARα in metabolic conversion of α-ESA into rumenic acid was studied, which might provide insights into potential enzymes participating in α-ESA metabolism.

## 2. Methods

### 2.1. Preparation of BMSO

Bitter melon seed (supplied by Hualien District Agricultural Research and Extension Station, Hualien, Taiwan) was powdered and agitated overnight in 10 volumes of *n*-hexane at room temperature. After filtration of the suspension through filter paper (Whatman No. 1), the residue was re-extracted using the same method and the filtrates were combined and evaporated under reduced pressure and used as BMSO. The yield of BMSO from 100 g of bitter melon seed was 28 g.

### 2.2. Animals and Diets

Homozygous PPARα-null male mice, on a pure C57BL/6J genetic background, and their wild-type control mice, were purchased from Jackson Lab (Bar Harbor, ME, USA). Mice were 6 weeks of age at the start of this study. After acclimation for 1 week, mice were fed a high-fat diet alone (30% soybean oil; HS) or containing BMSO (15% soybean oil + 15% BMSO; HB) for 5 weeks. The anti-adiposity and anti-steatosis effects of BMSO were compared to those of soybean oil, which has a PUFA concentration approximately equal to that of BMSO, to eliminate the perceivable benefits of PUFA (vs. SFA) on lipid metabolism. Diet composition (including fatty acids) are shown in Table S1. The BMSO dose used in this study was based on our previous studies [4,14], and a high dose was adopted to achieve functions within short time. All mice were kept in a room maintained at 23 ± 2 °C, with a controlled 12-h light:dark cycle and free access to feed and drinking water. Body weight was recorded weekly and food intake recorded every other day. Protocols for animal care and handling were approved by the Institutional Animal Care and Use Committee of China Medical University (protocol No. 101-40-N).

After 5 weeks of feeding, food was withheld overnight and the mice were killed by carbon dioxide asphyxiation. Liver and WAT (retroperitoneal, epididymal, and inguinal fat) were excised and weighed. Aliquots of liver and inguinal fat were quick-frozen in liquid nitrogen and stored at −80 °C for RNA extraction, and a small portion of liver and inguinal fat were frozen at −20 °C for chemical analysis. Remaining portions of the liver were freshly homogenized in 0.05 mol/L phosphate buffer (pH 7.4) for preparation of postnuclear supernatant, as described [16].

### 2.3. Measurement of Biochemical Indices

Liver lipids were extracted using a mixture of CHCl_3_/MeOH (2:1, *v*/*v*) and then Triton X-100 was added to solubilize the lipid extract, as described [16]. Lipid extracts were measured using Randox Laboratory commercial kits (Crumlin, Northland, UK) for cholesterol (CHOD-PAP) and triglyceride (GPO-PAP). Peroxisomal acyl-CoA oxidase (ACOX) activities in the postnuclear supernatant of liver were determined by the method of Small et al. [17].

### 2.4. Fatty Acid Analysis

Lipids extracted from liver and inguinal fat were subjected to transesterification by the sodium methoxide method [18] and the resulting fatty acid methyl esters were dissolved in *n*-hexane for fatty acid analysis in a Hewlett-Packard 5890 gas chromatograph using flame ionization detection on a DB-1 fused-silica capillary column (60 m × 0.25 mm × 0.1 μm, Agilent, Inc., Palo Alto, CA, USA) with nitrogen as carrier gas (1.5 mL/min). The oven temperature program was set at 60 °C for 2 min, then was increased at 10 °C/min to 170 °C, then at 3 °C/min to 270 °C, then held at 270 °C for 15 min. Fatty acid peaks were identified by comparison of retention times with those of reference standards.

### 2.5. RNA Isolation and mRNA Detection

Total RNA was extracted from homogenized liver or inguinal fat tissues using TRIzol reagent (Invitrogen, Carlsbad, CA, USA), according to manufacturer’s instructions. Total RNA (1 μg) was reverse-transcribed into first-strand cDNA using 200 units of Moloney murine leukemia virus reverse transcriptase (MMLV-RT) (Promega, Madison, WI, USA) in a total volume of 20 μL. For real-time PCR, a SYBR system with self-designed primers was used (Table S2). Amplification using 40 cycles of 2 steps (95 °C for 15 s and 60 °C for 1 min) was performed on an ABI Prism 7900HT sequence detection system.

### 2.6. Histology and In Situ Cell Death Detection

Inguinal fat fixed in 10% formalin was dehydrated through a graded ethanol series, embedded in paraffin, and cut into 5 μm sections. After staining with hematoxylin and eosin, sections were examined under a light microscope (OLYMPUS IX71) equipped with a SPOT RT color-2000 digital camera (Diagnostic Instruments, Sterling Heights, MI, USA). For uncoupling protein 1 (UCP1) immunostaining, sections were deparaffinized, rehydrated, and incubated with 0.5% Triton X-100, then blocked using 5% goat serum in phosphate-buffered saline (PBS). The primary antibody was rabbit antibody against human UCP1 (Abcam, Cambridge, UK), used at a dilution of 1:100 in PBS, whereas the secondary antibody was biotinylated goat anti-rabbit IgG antibody (Dako, Carpinteria, CA, USA), at a dilution of 1:250 in PBS. For detection of apoptosis, the TUNEL assay used terminal deoxynucleotidyl transferase and tetramethyl-rhodamine-dUTP (Roche Applied Science, Indianapolis, IN, USA) to directly label DNA strand breaks (red fluorescence). Images were acquired using a fluoromicroscope equipped with a SPOT RT color-2000 digital camera (Diagnostic Instruments, Sterling Heights, MI, USA).

### 2.7. Immunoblotting

Inguinal fat was homogenized in RIPA buffer containing 1% protease inhibitor cocktail and 1% phosphatase inhibitor cocktail (Sigma-Aldrich, St. Louis, MO, USA) and samples (60 μg of protein) were subjected to electrophoresis on 10% sodium dodecyl sulfate (SDS) gels, transferred to a polyvinylidene fluoride-plus transfer membrane (NEN Life Science, Boston, MA, USA), and immunoblotted. Primary antibodies were mouse antibody against bovine cellular retinol binding protein 1 (CRBP1) (Santa Cruz Biotechnology, Dallas, TX, USA) used at a dilution of 1:1000 in PBS, or rabbit antibodies against human swiprosin 1/Efhd2 (Sigma-Aldrich, St. Louis, MO, USA), human tyrosine hydroxylase (TH), human extracellular superoxide dismutase 3 (EC-SOD3), human uncoupling protein 1 (UCP1), or human cathepsin D (all from Abcam, Cambridge, UK), horse cytochrome c (from Santa Cruz), human light chain 3 (LC3), human acetyl-CoA carboxylase (ACC), rat phospho-ACC (Ser79), human PKA catalytic subunit, human phospho-PKA catalytic subunit (Thr197), human AMPK, or human phospho-AMPK (Thr172) (all from Cell Signaling, Danvers, MA, USA). Secondary antibodies were horseradish peroxidase (HRP)-labeled donkey anti-rabbit IgG or rabbit anti-mouse IgG (Amersham International, Amersham, UK) at a dilution of 1:5000 in PBS. Bound antibodies were detected using an enhanced chemiluminescence Western blotting kit (Amersham International) and images were quantified by densitometric analysis (Multimage Light Cabinet, Alpha Innotech Corporation, San Leandro, CA, USA).

### 2.8. Statistical Analysis

Data are expressed as mean ± SEM. A two-way ANOVA was used to determine significance of diet (HS vs. HB), genotype (WD vs. KO), and their interaction. When there was an interaction (*p* < 0.05) between diet and genotype, differences among four groups were located with Duncan’s multiple range test. Significant differences between two groups were analyzed by Student’s *t* test. If variances were not homogeneous, data were log-transformed before statistical analysis. The General Linear Model package (SAS Institute, Cary, NC, USA) was used for all statistical analyses, and differences were considered significant at *p* < 0.05.

## 3. Results

### 3.1. Effects of BMSO or PPARα Deficiency on Liver Lipid Content and Adiposity

During the experimental period, diet or genotype did not affect food intake or body weight gain, though the latter tended to be lowered by the HB diet (6.1 ± 0.8 vs. 4.3 ± 0.6 g/5 weeks for HS- and HB-fed mice, *p*-diet = 0.07). The body weight change during the experimental period is shown in Figure S1. At the endpoint, liver weight was constant among groups (1.1 ± 0.2 g) and PPARα deficiency significantly increased amounts of triglyceride and cholesterol in liver lipids (*p*-genotype < 0.05; Table 1). The HB lowered liver triglyceride (reduced by 28%) in WD mice, but had an elevating effect (increased by 130%) on KO mice (*p*-interaction < 0.05); therefore, the KO-HB group had significantly greater amounts of liver triglyceride than the KO-HS or WD-HS groups (all three were greater than the WD-HB group). In contrast to hepatic lipids, genotype had no effect on body fat content of retroperitoneal, epididymal, or inguinal fats, whereas the HB diet significantly and independently lowered the fat content (reduced by 50%, 35%, and 30%, respectively) of these three depots (*p*-diet < 0.05).

### 3.2. Effects of BMSO or PPARα Deficiency on Liver Lipid Metabolism

**Figure 1:**
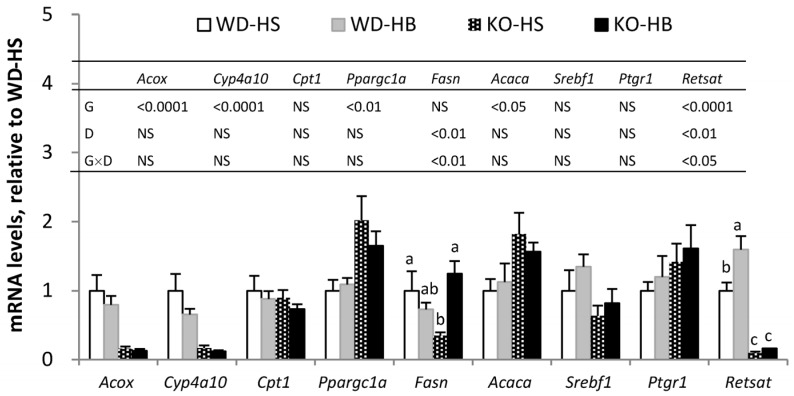
Hepatic mRNA levels of genes associated with lipid metabolism in WD and KO mice fed HS or HB diet for 5 weeks. Data are mean ± SEM, *n* = 8. Results of two-way ANOVA are shown in table (D, diet; G, genotype; D × G, interaction; NS, not significant). When there was a significant interaction between D and G, the significance of differences among groups was further analyzed by one-way ANOVA and Duncan’s multiple range test. ^a–c^ Values without a common superscript differed (*p* < 0.05).

For lipid metabolism in liver, mRNA levels of *Acox*, *Cyp4a10*, and *Cpt1*, which encode enzymes (peroxisomal acyl-CoA oxidase 1 and microsomal cytochrome P450 4A10) and a transporter (carnitine palmitoyltransferase I) participating in fatty acid catabolism, as well as *Fasn*, *Acaca* and *Srebf1*, which encode enzymes (fatty acid synthase and acetyl-CoA carboxylase 1) or transcription factor (sterol regulatory element-binding protein-1c) involving in lipogenesis, were measured. *Ppargc1a* encodes PPARγ coactivator 1-α, which is implicated in mitochondrial biogenesis [19]. As expected, ACOX activity (Table 1) and *Acox* and *Cyp4a10* transcripts (Figure 1), representing PPARα activity, were significantly suppressed in the absence of PPARα (*p*-genotype < 0.05). The HB diet increased ACOX activity in WD but not in KO mice (*p*-interaction < 0.05), as the value of group WD-HB was significantly greater than others. However, amounts of *Acox* and *Cyp4a10* mRNA were not affected by the HB diet. PPARα deficiency led to increased amounts of *Ppargc1a and Acaca* mRNA, regardless of diet. For *Fasn*, there was a significant interaction of genotype and diet, as groups KO-HB and WD-HS had values significantly greater than group KO-HS, whereas group WD-HB was intermediate.

### 3.3. Effects of BMSO or PPARα Deficiency on WAT Browning and Tissue Remodeling

**Figure 2:**
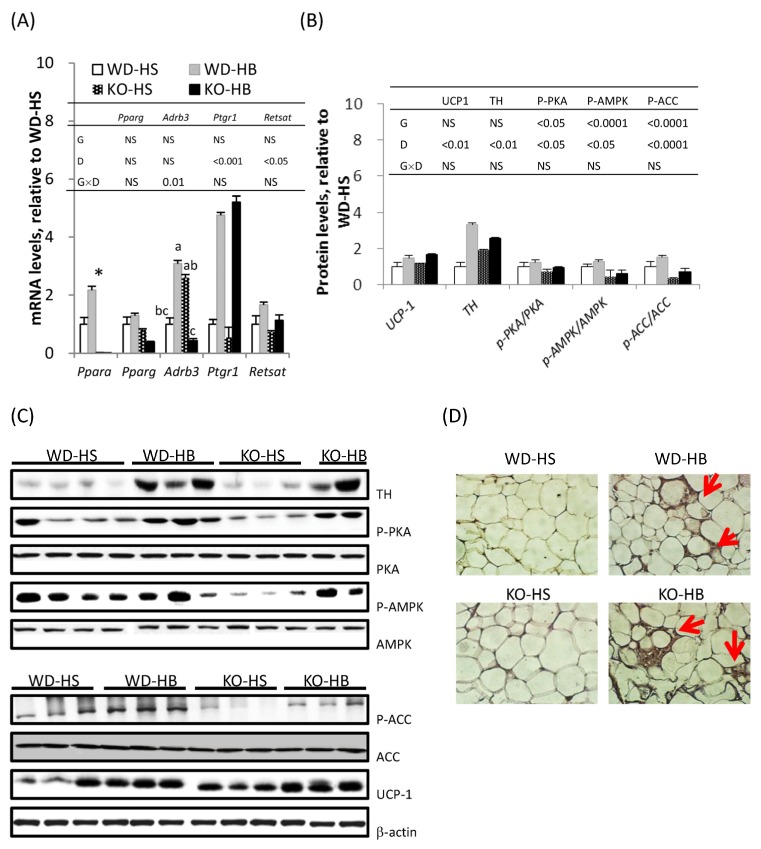
Levels of mRNA (**A**) and proteins (**B**) serve as markers involved in β-adrenergic stimulation and white adipose tissue (WAT) browning in the inguinal fat of WD and KO mice fed HS or HB diet for 5 weeks; (**C**) representative picture of immunoblot; (**D**) immunohistochemical staining of uncoupled protein 1 (UCP-1) in inguinal fat. In (**A**,**B**), data are mean ± SEM, *n* = 8. Results of two-way ANOVA are shown in the tables (D, diet; G, genotype; D × G, interaction; NS, not significant). When there was a significant interaction between D and G, the significance of differences among groups was further analyzed by one-way ANOVA and Duncan’s multiple range test. ^a–c^ Values without a common superscript differed (*p* < 0.05). * Different from WD-HS, *p* < 0.05 (by Student’s *t* test). In (**D**), brown fat-like cells are indicated with arrowheads.

As a master regulator for adipogenesis, PPARγ was abundantly and constantly expressed in the WAT (Figure 2A). Consistent with its absence in KO mice, *Ppara* mRNA was only detected in WD mice, and it was significantly increased by the HB diet. The protein encoded by *Adrb3* is a β-adrenoreceptor which mediates catecholamine-induced activation of adenylate cyclase through the action of PKA, and is involved in the regulation of lipolysis and thermogenesis in adipocytes [20]. When a comparison was made within genotype, the mRNA level of *Adrb3* was increased in WD mice, but decreased in KO mice fed the HB diet (*p*-interaction < 0.05).

Based on immunoblotting WAT UCP-1 and TH—proteins responsible for thermogenesis and catecholamine synthesis, respectively—were significantly increased by the HB diet, irrespective of genotype (Figure 2B,C). The HB diet also resulted in significantly greater phosphorylation levels of PKA, AMPK, and ACC proteins, regardless of genotype, whereas PPARα deficiency per se contributed to lowered phosphorylation levels of PKA, AMPK, and ACC proteins. The presence of brown fat-like cells in the WAT was apparent based on immunohistochemistry (IHC) staining of UCP-1 (Figure 2D). Positive UCP-1 staining and multilocular oil droplets were present in the cytoplasm of inguinal fat adipocytes from groups WD-HB and KO-HB, but barely detectable in adipocytes from groups WD-HS and KO-HS.

**Figure 3:**
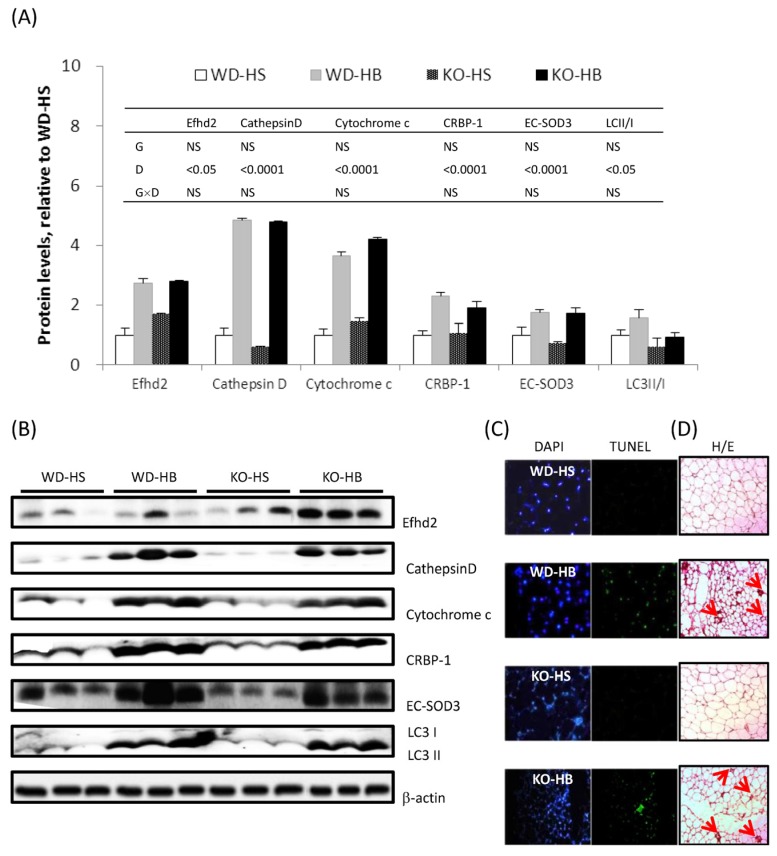
(**A**) Levels of proteins associated with WAT inflammation, browning, autophagy, and apoptosis in the inguinal fat of WD and KO mice fed HS or HB diet for 5 weeks; (**B**) representative picture of immunoblot; (**C**) TUNEL assay and (**D**) hematoxylin and eosin (H/E) staining of the inguinal fat. In (**A**), data are mean ± SEM, *n* = 8. Results of two-way ANOVA are shown in the tables (D, diet; G, genotype; D × G, interaction; NS, not significant). In (**C**), the same field is shown stained for total or apoptotic nuclei by DAPI or TUNEL, respectively; in (**D**), crownlike structure is indicated by arrowheads.

Proteins known to be upregulated by BMSO administration and associated with WAT delipidation, inflammation, browning, apoptosis, and autophagy were measured by immunoblotting (Figure 3A,B). Among them, Efhd2/swiprosin depends on a stabilized actin cytoskeleton that is required for immune responses [21]. Cathepsin D is a lysosomal protease closely linked to autophagy [22]. Cytochrome c and CRBP-1 serve as markers for mitochondria and preadipocyte numbers, respectively [23]. Antioxidative enzyme EC-SOD3 defends tissue from reactive oxygen species (ROS)-induced damage. LC3 II/I ratio serves as an indicator of autophagy. Irrespective of genotype, HB diet increased protein concentrations of Efhd2/swiprosin, cathepsin D, cytochrome c and CRBP-1, EC-SOD3, and the LC3 II/I ratio in inguinal fat. Based on TUNEL, there were apoptotic nuclei in the inguinal fat of both genotypes receiving HB, but not the HS diet (Figure 3C). Macrophage infiltration into WAT was evident based on crownlike structure (CLS) in groups WD-HB and KO-HB (Figure 3D).

### 3.4. Effects of BMSO or PPARα Deficiency on α-ESA Metabolism

For α-ESA conversion, mRNA amounts of *Ptgr1* and *RetSat* were measured in liver (Figure 1) and inguinal fat (Figure 2A). Transcripts of WAT *Ptgr1* and *RetSat*, as well as hepatic *RetSat*, were upregulated by the HB diet (*p*-diet < 0.05). PPARα deficiency significantly suppressed *RetSat* mRNA in liver but not in inguinal fat, leading to a significantly greater hepatic value for WD-HB than WD-HS group, with both significantly greater than the two KO groups (*p*-interaction < 0.05).

**Figure 4:**
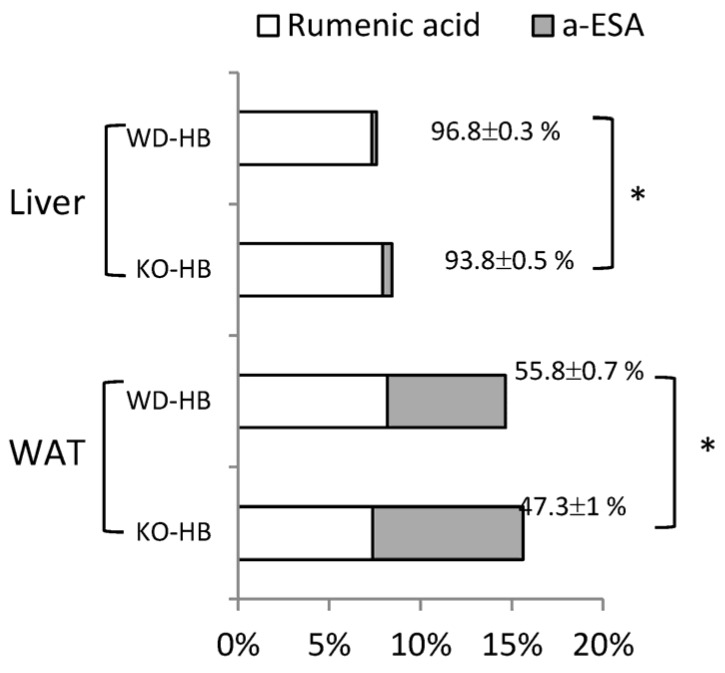
The rumenic acid and α-eleostearic acid (α-ESA) percentage of total fatty acids in lipids from liver and inguinal fat (WAT) of WD and KO mice fed the HB diet for 5 weeks. Conversion rate is calculated as (rumenic acid/(rumenic acid + α-ESA) × 100%) and expressed as mean ± SEM, *n* = 8. * Different from WD-HB, *p* < 0.05 (by Student’s *t* test).

The fatty acid composition in the liver and inguinal fat are shown (Tables S3 and S4, respectively). Though genotype and diet differentially and interactively affected tissue fatty acid profiles, we focus on α-ESA, from exogenous diet, and rumenic acid, from endogenous conversion. There was no *trans*-10, *cis*-12-CLA detected in either of the tissues. Accumulation of conjugated fatty acids (sum of α-ESA and rumenic acid), occurred only in the HB-treated groups, and was greater in the WAT than in the liver (Figure 4). Conversion of α-ESA into rumenic acid was almost 100% in liver, but only half of that in inguinal fat. In addition, the conversion rate in the KO mice, in both liver and WAT, was slightly but significantly lower than that in WD mice.

## 4. Discussion

This in vivo study, combined with our previous in vitro evidence [5,15], further elucidated the underlying mechanisms of impacts of BMSO or α-ESA on lipid homeostasis in liver and adipose tissues. The phenotype of this PPARα KO includes hepatic steatosis and spontaneous, late-onset obesity, with sexual dimorphism [24]. The present study used younger male mice; therefore, the extent of adiposity of KO mice did not differ from that of the WD mice. Ablation of PPARα in high-fat diet-fed mice eliminated anti-steatosis, but preserved the anti-adiposity function associated with BMSO administration, indicating that PPARα was required for BMSO-induced improvements in hepatic lipid metabolism/transportation, but was not essential for its effects on WAT regarding cellular repair/remodeling. Based on altered protein expression of Efhd2/swiprosin, cathepsin D, cytochrome c, CRBP-1, EC-SOD3, and ratio of LC3 II/I, combined with histological evidence, BMSO-featured WA—characterized by inflammation, browning, apoptosis, autophagy, repair and remodeling as evidenced in our previous report [14]—was reproduced in KO-HB mice.

Although the BMSO-mediated anti-steatosis effect disappeared without functional PPARα, hepatic PPARα activation by BMSO in this study was not as prominent as expected, since only enzyme activity, but not mRNA of the PPARα target gene, was increased by BMSO in WD mice. This might be ascribed to a long-term adaptive response. α-ESA, the major fatty acid in BMSO, has been recognized as a potent PPARα agonist compared to common fatty acids [11]. Using H4IIEC3, a PPARα-responsive hepatoma cell line, we demonstrated that α-ESA reduced intracellular triglyceride accumulation by increasing mRNA concentrations and enzyme activity of PPARα target genes after 24 or 48 h of treatment [5]. Moreover, α-ESA activated sirtuin 1 deacetylase and AMPK signaling, thus forming a feed-forward PPARα/AMPK/sirtuin 1 signaling loop, shifts hepatic lipid metabolism towards catabolism [5].

In accordance with our previous study [4,14], BMSO activated PKA signaling and increased thermogenic capacity of WAT in WD mice. An mRNA level of WAT *Ppara* was greater in WD-HB than WD-HS, which in line with the notion that PPARα is barely detectable unless WAT transforms its activity from fat storage into fat-burning [25]. However, PPARα was not required for this transformation, since with PPARα being ablated, activation of PKA and AMPK, as well as WAT browning, persisted in HB-treated mice. Transcriptional regulation of *Ucp1* involves PKA and p38 MAPK signaling, which phosphorylate CREB and ATF2, produce a coordinated network among PGC1α, PPARα, PPARγ, type 2 deiodinase, and thyroid hormone receptor, synergistically contributing to *Ucp1* transactivation [26,27]. Given that ablation of PPARα did not compromise cold-induced expression of UCPs in intrascapular brown adipose tissue, liver, and muscle [28] or UCP1 induction in retroperitoneal fat [27], many of the sites for regulating *Ucp1* expression might be redundant [29] and dispensable for PPARα, consistent with outcomes in the current study.

In KO mice, *Adrb3* was down- instead of upregulated by BMSO in normal controls; regardless, processes downstream of PKA activation was still detected. Thus far, we cannot explain why the HB diet had an opposite trend on *Adrb3* transcript, with or without PPARα, although other G-coupled proteins (or adrenaline/noradrenaline receptors) on WAT membranes might have enabled ADRB3 to transmit adrenergic innervation to PKA signaling.

α-ESA induced apoptosis in 3T3-L1 preadipocytes [15,30] and apoptotic nuclei were detected in the WAT of BMSO-fed mice (reference [4] and Figure 3 in current study). Likewise, CLA, specifically *trans*-10, *cis*-12 rather than the *cis*-9,*trans*-11 isomer, causes apoptosis in WAT [31] and at a lower dose (without causing lipoatrophy) or with short-term gavage, WAT browning and alternatively accumulation of activated M2 macrophages were present in the remaining fat depots [32,33]. CLA modulates body composition in a PPARα-independent manner, and a greater number of WAT UCP1 transcripts occurred in both WD and KO mice [34]. Therefore, α-ESA might act alike *trans*-10, *cis*-12-CLA on the WAT, whereas this tissue seems to be far more sensitive to *trans*-10, *cis*-12-CLA than to α-ESA, as the anti-adiposity dose needed for α-ESA (≥5%) [4] was much higher compared to CLA (0.1%~1%) in mouse-feeding trials [32,33]. Aside from cancer cells, we also determined that α-ESA- or *trans*-10, *cis*-12-CLA-induced apoptosis occurred exclusively in adipose tissue, where surplus and nonmetabolizable conjugated fatty acids accumulated.

CLS is frequently present in both obese and lipodystrophic WAT [35]. The coexistence of inflammation and browning in the WAT was expected, since cyclooxygenase 2 (COX2), a downstream effector of β-adrenergic signaling, is required for induction of brite cells [36]. Using targeted activation of programmed cell death in adipocytes, instead of classical M1, anti-inflammatory M2 macrophages were recruited into the remnant fat depot, which is apparently engaged in tissue repair and remodeling [37]. These M2 macrophages in the WAT can serve as a source of adrenaline and noradrenaline, in addition to sympathetic nerves, thus contributing to WAT browning [38]. We suspect the macrophages accumulated in the WAT of BMSO-treated mice might have an M2 phenotype, similar to those in CLA-fed mice [33]. Furthermore, that CLA-elicited insulin resistance and hepatic steatosis were absent in BMSO-treated mice [4], could be secondary responses to lipoatrophy, as a lack of adiponectin, rather than WAT inflammation, was the underlying cause of lipoatrophy-accompanied metabolic disturbance [35,39].

It is believed that α-ESA (or punicic acid) is converted into rumenic acid prior to further elongation, desaturation, and β-oxidation. We previously reported that the levels of *Ptgr1* and *RetSat* mRNA were upregulated in the H4IIEC3 cell line after 24 h incubation with α-ESA [5]. The current in vivo study further confirmed that transcripts of *Ptgr1* and *RetSat* in liver and WAT were upregulated in response to BMSO as a dietary source of α-ESA, supporting these two enzymes participated in this metabolic conversion. Liver was dominant in this conversion, relative to kidney, mucosa of the small intestine, and plasma [7]. In this study, based on fatty acid composition, there was complete conversion in liver but not in WAT (Figure 4). In that regard, WAT acquires fatty acids (from lipoprotein lipase) that act on chylomicrons carrying dietary lipids or very low-density lipoprotein (VLDL) carrying lipids exported from liver. The ability of WAT to conduct this conversion is unclear, although we detected rumenic acid in an α-ESA-treated 3T3-L1 cell culture [15], implying adipocytes per se were equipped with these metabolic enzymes. As each tissue has a distinct conversion ability, presumably the responsible enzymes also have tissue-specific distributions. In this study, the mRNA abundance of *RetSat* was much higher in liver than inguinal fat, although there was no such difference for the *Ptgr1* transcript (based on Ct values for qRT-PCR).

In this study, mice without PPARα were capable of converting α-ESA into rumenic acid, though less efficiently than their WD cohorts. It is noteworthy that the PPARα-regulated and starvation-induced gene (*Ppsig*) share the same open reading frame with *RetSat* and that it has been identified as a PPARα target gene [40]. The hepatic mRNA profile of *RetSat* in this study conformed to a typical PPARα-responsive gene, being markedly suppressed (basal levels) by PPARα deficiency, with induction by α-ESA only in WD mice. However, this PPARα-responsive pattern was absent in the WAT, perhaps due to low PPARα abundance in that tissue. In line with this, hepatic mRNA amounts of *RetSat/Ppsig* were reported to be upregulated in response to fasting or Wy-14643, two known PPARα activators, although there was no apparent induction in adipose tissue [40]. Although the current in vivo study highlighted the role of RETSAT in α-ESA metabolic conversion, it also indicated there were other PPARα-independent enzymes, presumably in addition to *PTGR1*, capable of conducting this saturation reaction.

## 5. Conclusions

BMSO-mediated anti-steatosis effect was PPARα-dependent, although this master regulator of lipid homeostasis was not essential for the anti-adiposity effect and α-ESA conversion of BMSO-fed mice. The PPARα-dependent enzyme RETSAT may participate in α-ESA conversion, although it has only a minor role.

## Figures and Tables

**Table 1:** Liver lipid content and acyl-CoA oxidase (ACOX) activity and body fat percentage of wild (WD) and knockout (KO) mice fed high-fat diet composed of soybean oil alone (HS) or with bitter melon seed oil (HB) for 5 weeks ^1^.

	Liver			Body Fat (%)		
	Triglyceride (mg)	Cholesterol (mg)	ACOX Activity (nmol/(min·mg Protein))	Retroperitoneal	Epididymal	Inguinal
WD-HS	12.2±1.2 ^b^	1.81 ± 0.19	2.62 ± 0.38 ^b^	0.87 ± 0.11	2.16 ± 0.27	1.11 ± 0.09
WD-HB	8.9 ± 1.1 ^c^	1.63 ± 0.14	4.05 ± 0.31 ^a^	0.39 ± 0.03	1.38 ± 0.15	0.75 ± 0.07
KO-HS	12.7 ± 0.7 ^b^	2.54 ± 0.30	1.92 ± 0.24 ^b^	0.66 ± 0.05	1.86 ± 0.09	1.12 ± 0.02
KO-HB	29.5 ± 8.8 ^a^	2.24 ± 0.17	2.46 ± 0.28 ^b^	0.36 ± 0.04	1.28 ± 0.09	0.86 ± 0.05
	***p*** **Values for Two-Way ANOVA**					
G	<0.01	<0.0001	<0.001	NS	NS	NS
D	NS	NS	<0.001	<0.0001	<0.01	<0.01
G × D	<0.05	NS	<0.05	NS	NS	NS

^1^ Data are means ± SEM, *n* = 8. Two-way ANOVA was conducted and results are shown in table (D, diet; G, genotype; D × G, interaction; NS, not significant). When there was a significant interaction between D and G, the significance of differences among groups was further analyzed by one-way ANOVA and Duncan’s multiple range test. ^a–c^ Values without a common superscript differed (*p* < 0.05).

## Supplementary Materials

The following are available online at http://www.mdpi.com/2072-6643/8/12/805/s1, Figure S1: Growth curve of mice fed experimental diet for 5 weeks, Table S1: Composition (%, by weight) of HS and HB diets used in this study, Table S2: Gene names and the sequences of the PCR primers, Table S3: Fatty acid composition of total lipids in the liver of WD and KO mice fed HS or HB diet for 5 weeks, Table S4: Fatty acid composition of total lipids in the inguinal fat of WD and KO mice fed HS or HB diet for 5 weeks.

## Abbreviations

ACC	acetyl-CoA carboxylase
α-ESA	α-eleostearic acid
AMPK	AMP-activated protein kinase
BMSO	bitter melon seed oil
CLA	conjugated linoleic acid
CLS	crown-like structure
CRBP1	cellular retinol binding protein 1
EC-SOD3	extracellular superoxide dismutase 3
KO-HB	PPARα knockout mice fed a high-fat diet composed of soybean oil plus BMSO
KO-HS	PPARα knockout mice fed a high-fat diet composed of soybean oil
LC3	light chain 3
NAFLD	nonalcoholic fatty liver disease
PKA	cAMP-dependent protein kinase
TH	tyrosine hydroxylase
WAT	white adipose tissue
WD-HB	wild-type mice fed a high-fat diet composed of soybean oil plus BMSO
WD-HS	wild-type mice fed a high-fat diet composed of soybean oil
