# Supplementary material for: Roles of Peroxisome Proliferator-Activated Receptor α in Bitter Melon Seed Oil-Corrected Lipid Disorders and Conversion of α-Eleostearic Acid into Rumenic Acid in C57BL/6J Mice
Source: Nutrients. 2016 Dec 12;8(12):805. doi: 10.3390/nu8120805 (PMC5188460; doi:10.3390/nu8120805)
Supplement: Supplementary file 1 [file nutrients-08-00805-s001.docx]

Supplementary Materials: Roles of Peroxisome Proliferator-Activated Receptor α in Bitter Melon Seed Oil-Corrected Lipid Disorders and Conversion of α-Eleostearic Acid into Rumenic Acid in C57BL/6J Mice

Ya-Yuan Chang, Hui-Min Su, Szu-Han Chen, Wen-Tsong Hsieh, Jong-Ho Chyuan
and Pei-Min Chao


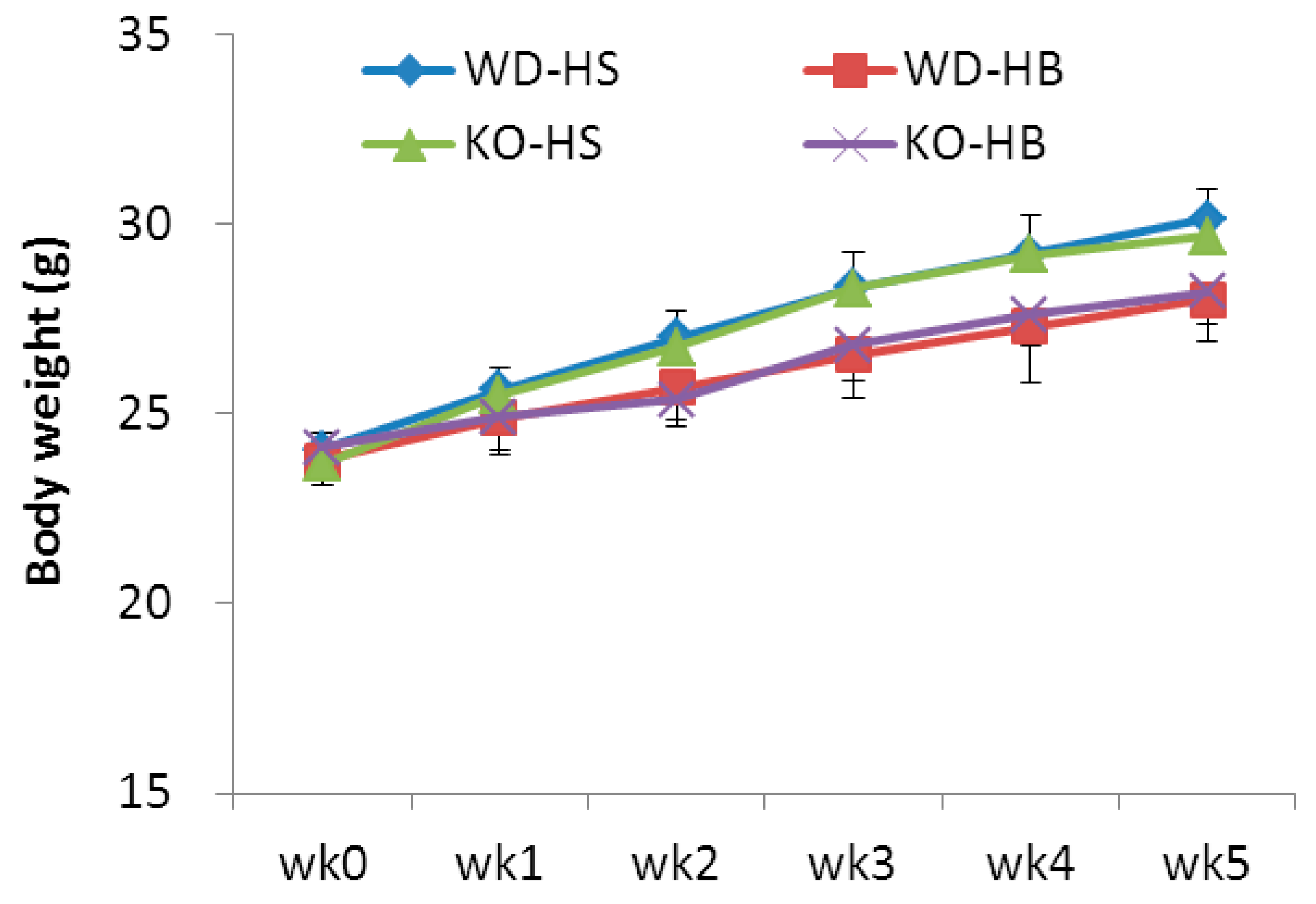


**Figure S1.** Growth curve of mice fed experimental diet for 5 weeks. Data are mean ± SEM, *n* = 8.

**Table S1.** Composition (%, by weight) of HS and HB diets used in this study.

|  | **HS** | **HB** |
| --- | --- | --- |
| Corn starch | 16.0 | 16.0 |
| Casein | 26.0 | 26.0 |
| Cellulose | 6.1 | 6.1 |
| Sucrose | 16.0 | 16.0 |
| Soybean oil ^1^ | 30.0 | 15.0 |
| BMSO ^2^ | -- | 15.0 |
| AIN-93 Mineral mixture | 4.2 | 4.2 |
| AIN-93 Vitamin mixture | 1.2 | 1.2 |
| dl-Cystine | 0.3 | 0.3 |
| Choline bitartrate | 0.2 | 0.2 |

^1^ The fatty acid composition of soybean oil: palmitic acid 11%, stearic acid 4%, oleic acid 22%, linoleic acid 56%, and linolenic acid 7%; ^2^ The fatty acid composition of BMSO: palmitic acid 4%, stearic acid 31%, oleic acid 4%, linoleic acid 9%, linolenic acid 1%, and α-ESA 51%.

**Table S2.** Gene names and the sequences of the PCR primers.

| **Gene** | **Encoding Protein** | **Accession Number** | **Primer** |
| --- | --- | --- | --- |
| *Acaca* | Acetyl-CoA carboxylase 1 | NM_133360.2 | F: ACCGGCTGAGTGATGGTGG  R: GGGAGCGCATTACAGACGG |
| *Acox* | Acyl-CoA oxidase | NM_015729 | F: CCAAGATTCAAGACAGAGCC  R: TCCCCTCAAGAAAATCCCC |
| *Adrb3* | β3-adrenergic receptor | NM_013462.3 | F: GCAGGAGGAAGATGGAAACCA  R: GAACCTGAGGCAACCCCTCT |
| *Cpt1* | Carnitine palmitoyltransferase I | NM_013495 | F: CCAAGATTCAAGACAGAGCC  R: TCCCCTCAAGAAAATCCCC |
| *Cyp4a10* | Cytochrome P450 4A10 | NM_010011.3 | F: TGAGGGAGAGCTGGAAAAGA  R: CTGTTGGTGATCAGGGTGTG |
| *Fasn* | Fatty acid synthase | NM_007988.3 | F: GCGGCTTCTGTGCCTGTTG  R: TCGGCAGCCCAGGCTAAGG |
| *Ppara* | PPARα | NM_001113418.1 | F: CAGCAACAACCCGCCTTTT  R: GCAGTGGAAGAATCGGACCTC |
| *Pparg* | PPARγ | NM_011146.3 | F: AAACTCTGGGAGATTCTCCTGTTG  R: GTGCTCATAGGCAGTGCATCA |
| *Ppargc1a* | PPARγ coactivator-1α | NM_008904.2 | F: AGCCGTGACCACTGACAACGAG  R: GCTGCATGGTTCTGAGTGCTAAG |
| *PTGR1*; *LTB4DH* | Leukotriene B4 12-hydroxydehydrogenase/15-ketoprostaglandin delta 13-reductase (LTB4 12-HD/PGR) | NM_008904.2 | F: CATCGTGAATCGGTGG  R: GCTAGGTCAAACGCAT |
| *RetSat* | Retinol saturase | NM_145084.1 | F: TCCATTCTGCCGAGCGTCTA  R: GGTGGTATGGCTGGGGGTTA |
| *Srebf1* | Sterol regulatory element binding transcription factor 1 | NM_001276707.1 | F: ACGAGCTACCCTTCGGTGA  R: TGTGTCTCCTGTCTCACCCC |

**Table S3.** Fatty acid composition of total lipids in the liver of WD and KO mice fed HS or HB diet for 5 weeks ^1,2^.

|  | **WD-HS** | **WD-HB** | **KO-HS** | **KO-HB** |
| --- | --- | --- | --- | --- |
|  | % total fatty acid weight | | | |
| C14:0 ^&^ | 0.25 ± 0.1 ^a,b^ | 0.27 ± 0.04 ^a,b^ | 0.31 ± 0.07 ^a^ | 0.21 ± 0.02 ^b^ |
| C16:0 *^,¥^ | 16.76 ± 0.76 | 14.88 ± 1.25 | 10.43 ± 0.30 | 9.25 ± 0.46 |
| C18:0 ^¥^ | 6.20 ± 1.02 | 9.37 ± 1.24 | 7.06 ± 1.04 | 10.23 ± 1.00 |
| C20:0 *^,¥,&^ | 0.09 ± 0.01 ^b^ | 0.14 ± 0.02 ^a^ | 0.16 ± 0.04 ^a^ | 0.13 ± 0.03 ^a^ |
| SFA | 23.32 ± 1.22 | 24.69 ± 1.42 | 18.04 ± 1.13 | 19.85 ± 1.18 |
| C16:1, *n*-9 * | 0.79 ± 0.09 | 0.70 ± 0.09 | 0.61 ± 0.04 | 0.63 ± 0.08 |
| C16:1, *n*-7 | 0.97 ± 0.31 | 1.31 ± 0.19 | 0.48 ± 0.21 | 0.72 ± 0.12 |
| C18:1, *n*-9 * | 13.6 ± 1.31 | 12.44 ± 0.83 | 17.46 ± 1.08 | 17.43 ± 1.22 |
| C18:1, *n*-7 | 0.95 ± 0.06 | 0.77 ± 0.05 | 1.19 ± 0.09 | 0.92 ± 0.16 |
| C20:1, *n*-9 *^,¥,&^ | 0.51 ± 0.03 ^a^ | 0.35 ± 0.04 ^c^ | 0.39 ± 0.03 ^b^ | 0.36 ± 0.02 ^c^ |
| MUFA | 16.92 ± 1.48 | 15.67 ± 1.06 | 20.27 ± 1.31 | 20.15 ± 1.43 |
| C18:2, *n*-6 *^,¥^ | 38.89 ± 1.35 | 31.52 ± 1.34 | 46.72 ± 1.08 | 38.67 ± 0.83 |
| C18:3, *n*-6 *^,¥,&^ | 1.10 ± 0.06 ^b^ | 1.04 ± 0.21 ^b^ | 1.47 ± 0.15 ^a^ | 0.86 ± 0.16 ^c^ |
| C20:2, *n*-6 *^,¥,&^ | 0.27 ± 0.03 ^b^ | 0.19 ± 0.02 ^c^ | 0.34 ± 0.01 ^a^ | 0.18 ± 0.04 ^c^ |
| C20:3, *n*-6 *^,¥^ | 0.61 ± 0.10 | 0.49 ± 0.07 | 0.29 ± 0.03 | 0.14 ± 0.04 |
| C20:4, *n*-6 *^,¥,&^ | 6.95 ± 0.70 ^b^ | 8.47 ± 1.00 ^a^ | 4.97 ± 0.67 ^c^ | 5.38 ± 0.48 ^c^ |
| C22:4, *n*-6 | 0.21 ± 0.03 | 0.20 ± 0.07 | 0.21 ± 0.13 | 0.14 ± 0.02 |
| *n*-6 PUFA | 48.10 ± 0.83 | 42.04 ± 0.99 | 54.07 ± 0.86 | 45.42 ± 0.54 |
| C18:3, *n*-3 *^,¥,&^ | 3.76 ± 0.41 ^a^ | 2.35 ± 0.31 ^c^ | 2.86 ± 0.31 ^b^ | 2.18 ± 0.17 ^c^ |
| C20:5, *n*-3 *^,¥,&^ | 0.87 ± 0.10 ^a^ | 0.46 ± 0.09 ^b^ | 0.32 ± 0.03 ^c^ | 0.28 ± 0.04 ^c^ |
| C22:5, *n*-3 *^,¥,&^ | 0.64 ± 0.07 ^a^ | 0.46 ± 0.09 ^b^ | 0.43 ± 0.08 ^b^ | 0.42 ± 0.05 ^b^ |
| C22:6, *n*-3 ^¥,&^ | 6.38 ± 0.55 ^a^ | 6.71 ± 0.54 ^a^ | 3.98 ± 0.36 ^b^ | 3.16 ± 0.55 ^c^ |
| *n*-3 PUFA | 11.64 ± 0.75 | 9.98 ± 0.32 | 7.59 ± 0.38 | 6.03 ± 0.56 |
| Rumenic acid ^¥^ | 0.01 ± 0.02 | 7.34 ± 0.42 | 0.00 ± 0.01 | 7.93 ± 0.32 |
| α-ESA ^¥^ | 0.00 ± 0.00 | 0.24 ± 0.05 | 0.00 ± 0.00 | 0.52 ± 0.05 |
| Conjugated FA | 0.01 ± 0.02 | 7.64 ± 0.45 | 0.00 ± 0.01 | 8.54 ± 0.35 |

^1^ Data are mean ± SEM, *n* = 8. * *p* < 0.05 for genotype, ^¥^ *p* < 0.05 for diet, ^&^ *p* < 0.05 for interaction. ^a–c^ Values without a common superscript differed (*p* < 0.05); ^2^ The detection limit ≥0.1%.

**Table S4.** Fatty acid composition of total lipids in the inguinal fat of WD and KO mice fed HS or HB diet for 5 weeks ^1,2^.

|  | **WD-HS** | **WD-HB** | **KO-HS** | **KO-HB** |
| --- | --- | --- | --- | --- |
|  | % total fatty acid weight | | | |
| C14:0 * | 0.53 ± 0.03 | 0.53 ± 0.09 | 0.41 ± 0.06 | 0.40 ± 0.02 |
| C16:0 *^,¥^ | 12.24 ± 0.82 | 9.17 ± 1.18 | 10.29 ± 0.49 | 7.51 ± 0.23 |
| C18:0 | 2.32 ± 0.17 | 3.00 ± 0.20 | 3.70 ± 0.46 | 4.58 ± 0.30 |
| C20:0 | 0.07 ± 0.01 | 0.11 ± 0.01 | 0.16 ± 0.21 | 0.14 ± 0.02 |
| SFA | 15.23 ± 0.93 | 13.24 ± 1.29 | 14.65 ± 0.83 | 12.70 ± 0.41 |
| C16:1, *n*-9 ^¥^ | 0.53 ± 0.07 | 0.67 ± 0.07 | 0.57 ± 0.04 | 0.62 ± 0.06 |
| C16:1, *n*-7 *^,¥^ | 2.23 ± 0.22 | 2.59 ± 0.43 | 0.79 ± 0.31 | 1.20 ± 0.18 |
| C18:1, *n*-9 ^¥^ | 27.48 ± 0.67 | 25.48 ± 0.53 | 27.29 ± 1.04 | 24.33 ± 0.87 |
| C18:1, *n*-7 ^&^ | 1.40 ± 0.16 ^a^ | 1.13 ± 0.07 ^b^ | 1.52 ± 0.15 ^a^ | 0.97 ± 0.21 ^c^ |
| C20:1, *n*-9 | 0.53 ± 0.03 | 0.42 ± 0.02 | 0.48 ± 0.04 | 0.37 ± 0.09 |
| MUFA | 32.28 ± 0.91 | 30.46 ± 0.69 | 30.76 ± 1.24 | 27.76 ± 0.96 |
| C18:2, *n*-6 *^,¥^ | 47.00 ± 0.96 | 38.24 ± 1.06 | 50.56 ± 0.80 | 40.62 ± 0.45 |
| C18:3, *n*-6 | 0.35 ± 0.08 | 0.25 ± 0.02 | 0.28 ± 0.05 | 0.25 ± 0.09 |
| C20:2, *n*-6 ^¥^ | 0.14 ± 0.03 | 0.05 ± 0.01 | 0.11 ± 0.05 | 0.03 ± 0.02 |
| C20:3, *n*-6 | 0.24 ± 0.03 | 0.10 ± 0.02 | 0.10 ± 0.02 | 0.41 ± 0.42 |
| C20:4, *n*-6 *^,¥^ | 0.38 ± 0.04 | 0.26 ± 0.05 | 0.21 ± 0.05 | 0.14 ± 0.04 |
| C22:4, *n*-6 | 0.04 ± 0.01 | 0.01 ± 0.01 | 0.02 ± 0.01 | 0.01 ± 0.01 |
| *n*-6 PUFA | 48.17 ± 0.95 | 38.93 ± 1.00 | 51.28 ± 0.77 | 41.47 ± 0.75 |
| C18:3, *n*-3 *^,¥,&^ | 3.82 ± 0.21 ^a^ | 2.50 ± 0.20 ^b^ | 3.11 ± 0.16 ^b^ | 2.40 ± 0.16 ^b^ |
| C20:5, *n*-3 | 0.05 ± 0.01 | 0.00 ± 0.01 | 0.01 ± 0.01 | 0.01 ± 0.01 |
| C22:5, *n*-3 *^,¥,&^ | 0.09 ± 0.02 ^a^ | 0.03 ± 0.02 ^b^ | 0.02 ± 0.01 ^b^ | 0.02 ± 0.01 ^b^ |
| C22:6, *n*-3 *^,¥,&^ | 0.31 ± 0.04 ^a^ | 0.12 ± 0.06 ^b^ | 0.11 ± 0.03 ^b^ | 0.05 ± 0.02 ^c^ |
| *n*-3 PUFA | 4.27 ± 0.25 | 2.64 ± 0.21 | 3.26 ± 0.18 | 2.47 ± 0.18 |
| Rumenic acid ^¥^ | 0.00 ± 0.01 | 8.17 ± 0.65 | 0.00 ± 0.01 | 7.37 ± 0.32 |
| α-ESA ^¥^ | 0.00 ± 0.00 | 6.47 ± 0.30 | 0.00 ± 0.00 | 8.24 ± 0.78 |
| Conjugated FA | 0.00 ± 0.01 | 14.73 ± 0.45 | 0.00 ± 0.01 | 15.69 ± 0.72 |

^1^ Data are mean ± SEM, *n* = 8. * *p* < 0.05 for genotype, ^¥^ *p* < 0.05 for diet, ^&^ *p* < 0.05 for interaction. ^a–c^ Values without a common superscript differed (*p* < 0.05); ^2^ The detection limit ≥0.1%.
